# Associations of Cord Blood Vitamin D and Preeclampsia With Offspring Blood Pressure in Childhood and Adolescence

**DOI:** 10.1001/jamanetworkopen.2020.19046

**Published:** 2020-10-05

**Authors:** Mingyu Zhang, Erin D. Michos, Guoying Wang, Xiaobin Wang, Noel T. Mueller

**Affiliations:** 1 Department of Epidemiology, Johns Hopkins Bloomberg School of Public Health, Baltimore, Maryland; 2 Welch Center for Prevention, Epidemiology, and Clinical Research, Johns Hopkins University, Baltimore, Maryland; 3 Ciccarone Center for the Prevention of Cardiovascular Disease, Johns Hopkins School of Medicine, Baltimore, Maryland; 4 Center on the Early Life Origins of Disease, Department of Population, Family and Reproductive Health, Johns Hopkins Bloomberg School of Public Health, Baltimore, Maryland; 5 Division of General Pediatrics & Adolescent Medicine, Department of Pediatrics, Johns Hopkins School of Medicine, Baltimore, Maryland

## Abstract

**Importance:**

Maternal preeclampsia may be one of the early risk factors for childhood and adolescence elevated blood pressure (BP). It is unknown whether the intergenerational association between maternal preeclampsia and offspring BP differs by cord blood vitamin D levels.

**Objective:**

To assess the associations between maternal preeclampsia and offspring systolic BP (SBP) across childhood and adolescence and to test whether these associations vary by cord blood 25-hydroxyvitamin D [25(OH)D] concentrations (a biomarker of in utero vitamin D status).

**Design, Setting, and Participants:**

This prospective cohort study analyzed 6669 SBP observations from 754 mother-child pairs from the Boston Birth Cohort, who were enrolled from December 1998 to June 2009. Data were analyzed from October 2019 to March 2020.

**Exposures:**

Physician-diagnosed maternal preeclampsia. Plasma 25(OH)D concentrations measured in cord blood samples collected at delivery.

**Main Outcomes and Measures:**

Repeated SBP measures between 3 and 18 years of age. The SBP percentile was calculated based on the 2017 American Academy of Pediatrics hypertension guidelines. Mean difference in SBP percentile in children born to mothers with vs without preeclampsia was compared across different cord blood 25(OH)D levels.

**Results:**

There were 6669 SBP observations from the 754 children; 50.0% were female and 18.6% were born preterm. Of the 754 mothers, 62.2% were Black and 10.5% had preeclampsia. Median cord blood 25(OH)D was 12.2 (interquartile range, 7.9-17.2) ng/mL. Maternal preeclampsia was associated with 5.34 (95% CI, 1.37-9.30) percentile higher SBP after adjusting for confounders. This association varied by quartiles of cord blood 25(OH)D concentrations: the differences in SBP percentile comparing children born to mothers with vs without preeclampsia were 10.56 (95% CI, 2.54-18.56) for quartile 1 (lowest), 7.36 (95% CI, –0.17 to 14.88) for quartile 2, 4.94 (95% CI, –3.07 to 12.96) for quartile 3, and –1.87 (95% CI, –9.71 to 5.96) for quartile 4 (highest). When cord blood 25(OH)D was analyzed continuously, children born to mothers with preeclampsia had 3.47 (95% CI, 0.77-6.18) percentile lower SBP per 5 ng/mL 25(OH)D increment. These associations did not differ by child sex or developmental stages.

**Conclusions and Relevance:**

In this study of a US high-risk birth cohort, maternal preeclampsia was associated with higher offspring SBP from early childhood to adolescence. These associations were attenuated by higher cord blood 25(OH)D levels in a dose-response fashion. Additional studies, including clinical trials, are warranted.

## Key Points

**Question:**

What is the association of in utero exposure to preeclampsia with blood pressure in childhood and adolescence, and does the association differ by cord blood vitamin D levels?

**Findings:**

In this cohort study of 754 mother-child pairs with 6669 blood pressure observations, maternal preeclampsia was associated with higher offspring systolic blood pressure from early childhood to adolescence. This association, however, was attenuated among children with higher cord blood 25-hydroxyvitamin D levels (reflecting in utero vitamin D status).

**Meaning:**

Results of this study suggest that optimizing maternal prenatal vitamin D levels may help prevent the development of high blood pressure in children born to mothers with preeclampsia.

## Teaser

This cohort study assesses the associations between maternal preeclampsia and offspring systolic blood pressure across early childhood, middle childhood, and adolescence and investigates whether the association varies by cord blood plasma vitamin D level.

## Introduction

Preeclampsia is the leading cause of maternal and perinatal mortality and morbidity, complicating 2% to 8% of pregnancies worldwide.^1^ From 1987 to 2004, the incidence rate of preeclampsia has increased by 24.6% in the US.^2^ Simultaneously, the prevalence of childhood elevated blood pressure (BP) has increased in the US by 39% from 1988 to 2008.^3^ High childhood BP is associated with hypertension and cardiovascular diseases in adulthood.^4,5^ Identifying antecedents of high childhood BP may thus help to alleviate the global burden of cardiovascular diseases.

Maternal preeclampsia may be one of the earliest risk factors for offspring elevated BP in childhood and adolescence. A meta-analysis of 44 293 mother-child dyads from 10 studies found that children born to mothers with preeclampsia have on average 2.4 mm Hg higher systolic BP (SBP).^6^ Most studies, however, measured offspring BP at a single point, making it difficult to understand how this association tracks across early child developmental stages. Furthermore, few of these studies evaluated whether preeclampsia affects childhood and adolescent BP differentially by sex, despite studies suggesting that preeclampsia may affect the fetus in a sexually dimorphic fashion.^7,8^

Vitamin D deficiency has been associated with an increased risk of cardiovascular diseases including preeclampsia.^9^ In a recent meta-analysis of 27 randomized clinical trials with 4777 participants, vitamin D supplementation in pregnancy was associated with 0.37 times the odds of developing preeclampsia vs control.^10^ Vitamin D also plays a role in fetal development^11^ and higher early-life vitamin D levels may be protective against childhood high BP.^12,13,14^ However, to our knowledge, no study has examined whether the intergenerational association of preeclampsia with childhood and adolescent BP varies by vitamin D status.

In this study, we aimed to assess the associations between maternal preeclampsia and offspring SBP across developmental stages (early childhood [ages 3-5 years], middle childhood [ages 6-12 years], and adolescence [ages 13-18 years]) and to examine whether this association differs by cord blood vitamin D level.

## Methods

### Study Participants

We used data from the Boston Birth Cohort, an ongoing prospective birth cohort that has been recruiting mother-infant pairs since 1998 from the Boston Medical Center, Boston, Massachusetts. Detailed methods of recruitment have been published previously.^15^ Mothers were recruited 24 to 72 hours after delivery. Multiple gestation pregnancies and neonates with major birth defects were excluded from recruitment. Eligible mothers who consented to participate in this study were interviewed through administration of a standardized postpartum questionnaire that collected mother’s sociodemographic information.

This analysis included 754 mother-child pairs of the Boston Birth Cohort who were enrolled from December 1998 to June 2009 and received pediatric primary care at the Boston Medical Center. We excluded pairs who did not have data on maternal preeclampsia diagnosis, cord blood 25-hydroxyvitamin D (25(OH)D), or child BP from 3 to 18 years of age. A diagram of the participant inclusion and exclusion is shown in eFigure 1 in the Supplement. The follow-up period of this analysis was from February 2002 to May 2018. Data were analyzed from October 2019 to March 2020.

The study was approved by the institutional review boards of the Boston Medical Center and the Johns Hopkins Bloomberg School of Public Health. All mothers provided written informed consent for participation in the study. This study followed the Strengthening the Reporting of Observational Studies in Epidemiology (STROBE) reporting guideline for cohort studies.

### Maternal Preeclampsia

We extracted physician diagnosed maternal preeclampsia data from the electronic medical records. At the time when mothers in this cohort were pregnant, preeclampsia diagnosis was based on the American College of Obstetricians and Gynecologists 2002 guideline (new onset of SBP ≥140 mm Hg or diastolic BP ≥90 mm Hg after 20 weeks of gestation plus having proteinuria).^16^

### Childhood and Adolescence SBP

Child SBP was measured between 3 and 18 years of age. Clinical staff measured BP using a validated automatic sphygmomanometer (Masimo SET; Masimo Corp) with an appropriately sized cuff at the right brachial artery in a quiet room with the child in a sitting position.^17^ We calculated child age-, sex-, and height-specific SBP percentile based on the 2017 American Academy of Pediatrics hypertension guidelines.^18^

### Cord Blood Vitamin D Concentrations

Cord blood samples were collected at delivery. We measured concentrations of 25(OH)D_2_ and 25(OH)D_3_ in cord blood plasma using high-performance liquid chromatography tandem mass spectrometry (HPLC-MS/MS) assay^19^ and summed the values to derive total 25(OH)D.

We modeled 25(OH)D as a continuous variable, as a categorical variable (by quartiles), and as a binary variable (vitamin D deficient vs not). We defined vitamin D deficiency as cord blood 25(OH)D less than less than 11 ng/mL (to convert to nanomoles per liter, multiply by 2.496) based on the Institute of Medicine Recommendations (1997)^20^ and consistent with previous studies.^12,21,22^ In a sensitivity analysis, we defined vitamin D deficiency as 25(OH)D less than 20 ng/mL based on the Institute of Medicine Dietary Reference Intakes^23^ which suggested that this level of plasma 25(OH)D meets the needs of approximately 97.5% of the population for bone health.

### Covariates

Maternal race/ethnicity, educational level, smoking status during pregnancy, and prepregnancy body mass index (BMI, calculated as weight in kilograms divided by height in meters squared) were obtained from the postpartum questionnaire. We defined maternal underweight as BMI less than 18.5, normal weight as BMI 18.5 to 25, overweight as BMI 25 to 30, and obese as BMI 30 or greater. We extracted maternal age at delivery and child birth weight, gestational age, and sex from the electronic medical records. We defined preterm birth as gestational age less than 37 weeks and low birth weight as birth weight less than 2500 g. A subsample of children (n = 586) had postnatal 25(OH)D concentration measured using HPLC-MS/MS assay (median [interquartile range] age at measurement, 1.27 [0.82-3.02] years).

### Statistical Analysis

We used linear mixed models (random intercepts for each mother-child pair and fixed effects for other covariates) to estimate the associations between maternal preeclampsia and repeated measurements of child SBP percentile. We examined whether the maternal preeclampsia–child SBP association varied by cord blood 25(OH)D level by including a product term of preeclampsia and cord blood 25(OH)D concentration. We visualized the associations between cord blood 25(OH)D and child SBP percentile across all developmental stages by maternal preeclampsia using a fractional polynomial prediction plot. In the subsample with postnatal 25(OH)D concentration measured, we further examined whether postnatal 25(OH)D confounded or modified the association of maternal preeclampsia and child SBP and whether postnatal 25(OH)D was associated with child SBP among those born to mothers with preeclampsia.

We defined confounders as covariates associated with both the exposure (maternal preeclampsia) and the outcome (child SBP) and not in the potential causal pathway.^24^ We adjusted for confounders including maternal age at delivery (continuous), race/ethnicity (Black; White; Hispanic; others), educational level (less than high school; high school graduate/general educational development; college graduate or above), smoking status during pregnancy (never, quitted, or continued), and prepregnancy BMI (underweight, normal weight, overweight, or obese). Given the reported seasonal variations of 25(OH)D levels within individuals,^25^ we additionally adjusted for season of delivery (Winter: December to February; Spring: March to May; Summer: June to August; Autumn: September to November) in a sensitivity analysis. We coded missing values for categorical variables as a separate category. There were no missing values for the continuous variable maternal age at delivery. In a sensitivity analysis, we used the multiple imputation by chained equations method^26^ to account for missing data.

We examined whether the associations differed by potential effect modifiers including child developmental stage (early childhood; middle childhood; adolescence), child sex (male/female), maternal race/ethnicity (Black; Hispanic), preterm birth (yes/no), low birth weight (yes/no), and maternal overweight or obese (yes/no) in the confounder-adjusted models. We included a 3-way interaction term of maternal preeclampsia, cord blood 25(OH)D concentration, and the potential effect modifier and used Wald test to test for the significance of the interaction term.

We compared the baseline characteristics of the 754 mother-child pairs included in this analysis with the 223 pairs excluded due to missing child SBP. To account for potential selection bias, we used the stabilized inverse probability weighting method as a sensitivity analysis.^27^ We estimated the probability of having missing child SBP and being excluded from the analysis based a set of baseline covariates (maternal age at delivery, race/ethnicity, educational level, marital status, smoking status during pregnancy, prepregnancy BMI, preterm birth, and low birth weight) and applied the weights to the regression models that adjusted for potential confounders.

We conducted all analyses using Stata version 15.1 (StataCorp). We considered a 2-sided *P* < .05 as statistically significant.

## Results

The analytic data set included 6669 SBP observations from 754 children age 3 to 18 years. The median number of SBP measurements per child was 7.0 (interquartile range, 4.0-11.0). A total of 672 (89.1%) children had SBP measurements in early childhood, 650 (86.2%) in middle childhood, and 143 (19.0%) in adolescence, contributing to 1753, 4265, and 651 SBP measurements, respectively.

The Table presents the characteristics of the mother-child pairs overall and by maternal preeclampsia and cord blood 25(OH)D concentration. Of mothers, 79 (10.5%) had preeclampsia during pregnancy, 469 (62.2%) were Black, 219 (29.0%) did not finish high school education, and 396 (52.5%) were overweight or obese. Of children, 377 (50.0%) were female, 140 (18.6%) were born preterm, and 120 (15.9%) had low birth weight. Median cord blood 25(OH)D concentration was 12.2 (interquartile range, 7.9-17.2) ng/mL; 324 (43.0%) and 626 (83.0%) children had cord blood 25(OH)D less than 11 ng/mL and 20 ng/mL, respectively. eTable 1 in the Supplement shows the characteristics of mother-child pairs by maternal preeclampsia and eTable 2 in the Supplement shows characteristics by quartiles of cord blood 25(OH)D concentration. Mothers who had preeclampsia had higher prepregnancy BMI (mean, 29.7 vs 26.6) and were more likely to have children born preterm (48.1% vs 15.1%) and low birth weight (43.0% vs 12.7%). Mothers with lower cord blood 25(OH)D concentrations were younger, more likely to be Black, had lower educational level, and were less likely to be married.

**Table.** Characteristics of the 754 Mother-Child Pairs in This Analysis, Overall and by Maternal Preeclampsia and Cord Blood 25(OH)D Concentration^a^

Characteristic	Overall	No preeclampsia (n = 675)		Preeclampsia (n = 79)	
		25(OH)D ≥11 ng/mL	25(OH)D <11 ng/mL	25(OH)D ≥11 ng/mL	25(OH)D <11 ng/mL
No.	754	383	292	47	32
Maternal characteristic					
Age at delivery, mean (SD), y	28.7 (6.7)	29.1 (6.8)	27.8 (6.5)	31.8 (6.5)	27.1 (5.6)
Black, No. (%)	469 (62.2)	206 (53.8)	209 (71.6)	29 (61.7)	25 (78.1)
Married, No. (%)	251 (33.3)	137 (35.8)	87 (29.8)	18 (38.3)	9 (28.1)
Did not finish high school, No. (%)	219 (29.0)	118 (30.8)	80 (27.4)	11 (23.4)	10 (31.2)
Smoked during pregnancy, No. (%)	76 (10.1)	36 (9.4)	28 (9.6)	8 (17.0)	4 (12.5)
Prepregnancy BMI, mean (SD)	26.9 (6.3)	26.2 (5.7)	27.1 (6.9)	29.9 (6.5)	29.4 (6.1)
Overweight or obesity, No. (%)	396 (52.5)	185 (48.3)	154 (52.7)	36 (76.6)	21 (65.6)
Child characteristic					
No. of blood pressure measurements, median (IQR)	7.0 (4.0-11.0)	7.0 (4.0-11.0)	7.0 (4.0-11.0)	7.0 (3.0-11.0)	8.5 (4.0-11.5)
Cord blood 25(OH)D concentration, median (IQR), ng/mL	12.2 (7.9-17.2)	16.5 (13.6-20.9)	7.3 (5.5-9.0)	15.2 (12.5-21.1)	7.3 (5.5-8.6)
Female, No. (%)	377 (50.0)	192 (50.1)	142 (48.6)	24 (51.1)	19 (59.4)
Gestational age, mean (SD), wk	38.5 (2.5)	38.6 (2.5)	38.8 (2.0)	36.5 (3.2)	37.1 (2.2)
Preterm birth, No. (%)	140 (18.6)	66 (17.2)	36 (12.3)	25 (53.2)	13 (40.6)
Birth weight, mean (SD), g	3103.8 (650.8)	3143.4 (643.7)	3163.2 (564.0)	2678.4 (920.1)	2713.9 (676.3)
Low birth weight, No. (%)	120 (15.9)	58 (15.1)	28 (9.6)	23 (48.9)	11 (34.4)

Abbreviations: BMI, body mass index (calculated as weight in kilograms divided by height in meters squared); 25(OH)D, 25-hydroxyvitamin D; IQR, interquartile range.

SI conversion factor: To convert 25(OH)D to nanomoles per liter, multiply by 2.496.

^a^ Characteristics of the mother-child pairs by maternal preeclampsia is provided in eTable 1 in the Supplement. Characteristics of the mother-child pairs by cord blood 25(OH)D concentration is provided in eTable 2 in the Supplement.

**Figure 1.**
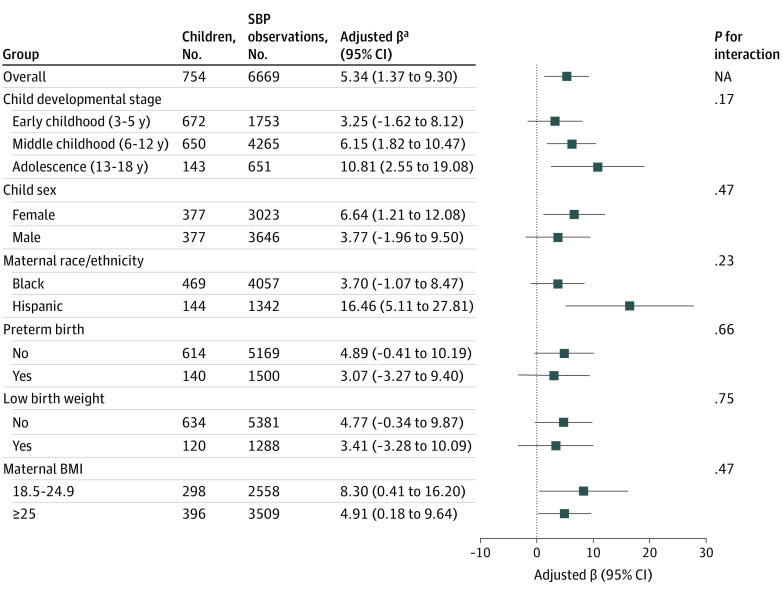
Mean Difference in Systolic Blood Pressure Percentile in Children Born to Mothers With Preeclampsia vs Mothers Without Preeclampsia, Overall and by Subgroup BMI indicates body mass index (calculated as weight in kilograms divided by height in meters squared); NA; not available; SBP, systolic blood pressure. ^a^Models were adjusted for maternal age at delivery, race/ethnicity (if not stratified by race/ethnicity), educational level, smoking status during pregnancy, and maternal prepregnancy BMI (if not stratified by maternal BMI).

Compared with children born to mothers without preeclampsia, those born to mothers with preeclampsia had 5.34 (95% CI, 1.37-9.30) percentile higher SBP after adjusting for confounders. This association did not differ by child developmental stage, sex, maternal race/ethnicity, preterm birth, low birth weight, or maternal overweight or obese status (Figure 1) (eTable 3 in the Supplement). The associations between maternal preeclampsia and child SBP did not change after further adjusting for season of delivery or when using the multiple imputation by chained equations method to account for missing data (eTable 3 in the Supplement).

The association between maternal preeclampsia and child SBP varied by cord blood 25(OH)D concentration (*P* = .007 for interaction between maternal preeclampsia and cord blood 25[OH]D on child SBP). The association between maternal preeclampsia and child SBP was stronger among those who had vitamin D deficiency (cord blood 25[OH]D <11 ng/mL) (adjusted β, 7.73; 95% CI, 1.60-13.86) compared with those who did not (adjusted β, 3.71; 95% CI, –1.46 to 8.87). When defining vitamin D deficiency as cord blood 25(OH)D less than 20 ng/mL, the association between maternal preeclampsia and child SBP percentile was 7.49 (95% CI, 3.13-11.85) for those with vitamin D deficiency and −4.19 (95% CI, −13.26 to 4.88) for those without vitamin D deficiency (eTable 4 in the Supplement).

**Figure 2.**
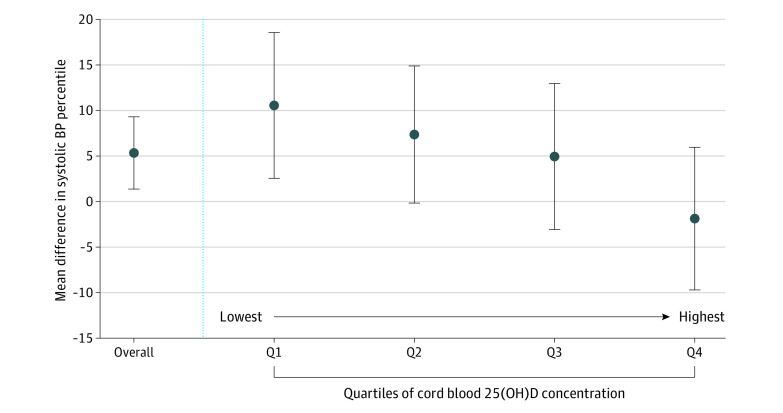
Mean Difference in Systolic Blood Pressure Percentile in Children Born to Mothers With Preeclampsia vs Mothers Without Preeclampsia, Overall and by Quartile (Q) of Cord Blood 25-Hydroxyvitamin D (25[OH]D) Concentration The quartiles of cord blood 25(OH)D concentration were Q1, 1.4-7.9 ng/mL; Q2, 8.0-12.2 ng/mL; Q3, 12.3-17.1 ng/mL; and Q4, 17.2-73.5 ng/mL (to convert to nanomoles per liter, multiply by 2.496). Models were adjusted for maternal age at delivery, race/ethnicity, educational level, smoking status during pregnancy, and maternal prepregnancy body mass index. Point estimates and corresponding 95% CIs are in eTable 4 in the Supplement.

Associations between maternal preeclampsia and child SBP changed in a dose-dependent manner by level of cord blood 25(OH)D concentration (Figure 2) (eTable 4 in the Supplement). By quartiles of cord blood 25(OH)D, the adjusted difference in SBP percentile comparing children born to mothers with vs without preeclampsia were 10.56 (95% CI, 2.55-18.56) for quartiles 1 (lowest), 7.36 (95% CI, –0.17 to 14.88) for quartile 2, 4.94 (95% CI, –3.07 to 12.96) for quartile 3, and –1.87 (95% CI, –9.71 to 5.96) for quartile 4 (highest). Associations also did not change after further adjusting for season of delivery or when using the multiple imputation by chained equations method to account for missing data (eTable 4 in the Supplement).

**Figure 3.**
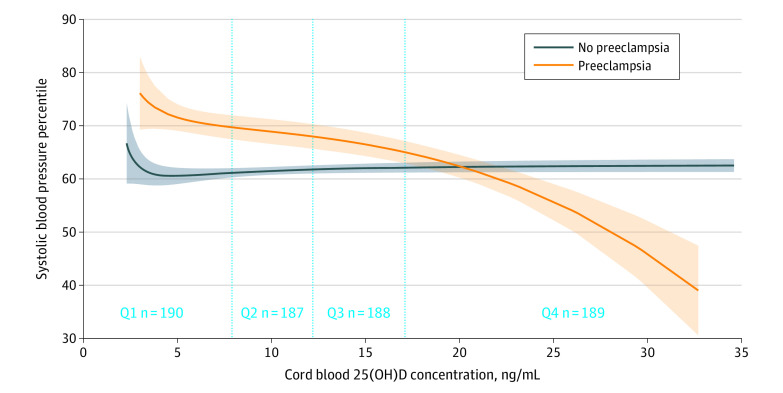
Fractional Polynomial Prediction Plot Showing the Association Between Cord Blood 25-Hydroxyvitamin D (25[OH]D) Concentration and Child Systolic Blood Pressure Percentile Observations with cord blood 25(OH)D concentration <1 percentile or >99 percentile were excluded. Shaded areas indicate 95% CIs. Q indicates quartile.

The fractional polynomial prediction plot showed the association of cord blood 25(OH)D concentration with child SBP percentile across all developmental stages by maternal preeclampsia (Figure 3). For children born to mothers with without preeclampsia, their SBP decreased monotonically with higher cord blood 25(OH)D concentration. There was no association of cord blood 25(OH)D concentration and child SBP for those born to mothers without preeclampsia. After adjustment for confounders, children born to mothers with preeclampsia had 3.47 (95% CI, 0.77-6.18) percentile lower SBP per 5 ng/mL increment in cord blood 25(OH)D. There was no association between cord blood 25(OH)D and SBP among children born to mothers without preeclampsia (0.37 [95% CI, –0.44 to 1.18] percentile increase in SBP per 5 ng/mL increment in cord blood 25[OH]D).

The possible modifying role of cord blood 25(OH)D level in the maternal preeclampsia—child SBP association did not differ by maternal race/ethnicity, preterm birth, low birth weight, or maternal overweight or obese status (eFigure 2 in the Supplement). However, we did observe a stronger decrease in child SBP with incrementing cord blood 25(OH)D level during adolescence (compared with early and middle childhood) and for female children (compared with male children).

In the subsample of children (n = 586) with postnatal plasma 25(OH)D measured, postnatal 25(OH)D did not confound or modify the association of maternal preeclampsia and child SBP (eTable 5 in the Supplement). Among those born to mothers with preeclampsia, postnatal 25(OH)D concentration was not associated with SBP (adjusted β for change in SBP percentile per 5 ng/mL increment in postnatal 25(OH)D, –1.17; 95% CI, –2.87 to 0.53).

eTable 6 in the Supplement shows the characteristics of the mother-child pairs included in vs excluded from the analysis due to missing child SBP. Mothers included were more likely to be Black (469 [62.2%]) and had higher prepregnancy BMI (mean [SD], 26.9 [6.3]); and their children were less likely to have been born preterm (140 [18.6%]). Results did not change after we applied the stabilized inverse probability weights in the regression models. In weighted models that adjusted for potential confounders, maternal preeclampsia was associated with 5.78 (95% CI, 1.99-9.58) percentile higher child SBP; the adjusted difference in SBP percentile comparing children born to mothers with vs without preeclampsia were 11.01 (95% CI, 4.57-17.46) for cord blood 25(OH)D quartile 1 (lowest), 7.66 (95% CI, 0.92-14.39) for cord blood 25(OH)D quartile 2, 5.40 (95% CI, –1.69 to 12.49) for cord blood 25(OH)D quartile 3, and –1.34 (95% CI, –9.83 to 7.15) for cord blood 25(OH)D quartile 4 (highest). Results for other models are shown in eTable 3 and eTable 4 in the Supplement.

## Discussion

In this prospective birth cohort of predominately urban, low-income, minority mother-child pairs from Boston, Massachusetts, we found that maternal preeclampsia was associated with higher child SBP from childhood to adolescence. This association, however, varied by cord blood 25(OH)D level, such that in children born to mothers with preeclampsia, higher cord blood 25(OH)D was associated with lower child SBP.

Our findings are consistent with previous studies of maternal preeclampsia and higher childhood and adolescence BP,^28,29,30,31,32,33,34,35,36^ although some did not find such associations^37,38,39,40^ or found the associations attenuated after adjusting maternal BMI.^41^ Most of these previous studies, however, focused on a specific child age or developmental stage and thus do not examine the association of preeclampsia with child BP across developmental stages. Our study findings suggest that the association between maternal preeclampsia and child SBP presents in both male and female participants, and persists from early childhood to late adolescence, independent of maternal BMI. Additionally, the inverse association of cord blood 25(OH)D and child SBP among children born to mothers with preeclampsia appears to be stronger in female than male participants. This finding merits further investigation given previous reports that the association between preeclampsia and child BP may be sex-specific^28^ and recent evidence showing that women are disproportionally affected by cardiovascular disease risk factors.^42,43^

Several possible mechanisms may be factors in the association between maternal preeclampsia and child BP. Fetal origins of cardiovascular pathology start in utero.^44^ Animal and human studies have shown that maternal preeclampsia is associated with offspring vascular and cardiac abnormalities, and higher inflammation and oxidative stress.^45^ Some of these associations may be sexually dimorphic.^7,8^ In a sibling study, Jayet et al^46^ found that children born to mothers with preeclampsia had higher pulmonary artery pressure and lower flow-mediated dilation compared with their siblings born when the mother did not have preeclampsia. Other possible explanations include shared genetic or familial environmental and lifestyle characteristics. Preeclampsia has a heritability at 55%-60%.^47^ The genetic contribution to preeclampsia has been confirmed in studies and multiple candidate genes linked to preeclampsia have been identified.^48^ This genetic predisposition to preeclampsia may be inherited by the offspring and cause higher BP.

Vitamin D may be associated with cardiovascular health and cardiovascular physiology and pathology through multiple pathways, as noted by Normal et al^49^ and Al Mheid el al,^50^ which include regulations of myocyte proliferation and hypertrophy^51^ and the renin-angiotensin system.^52^ Vitamin D also may be a factor in implantation, placentation, and angiogenesis processes and is essential for maintaining a healthy pregnancy.^53,54^ Mechanisms on how vitamin D may modify the association between preeclampsia and child BP, however, is unclear. Our findings need to be replicated in other observational studies. whether replicated, this benefit also needs to be confirmed in future clinical trials of vitamin D supplementation in pregnancies with preeclampsia with long term follow-up of their children. This cautious interpretation of our study findings owes to the inconclusive findings of randomized clinical trials examining how vitamin D supplementation may reduce adult BP.^50^

### Strengths and Limitations

This study has several strengths. First, we were able to examine the association of maternal preeclampsia with offspring SBP from early childhood to adolescence, and simultaneously evaluate the possible modifying role of cord blood vitamin D. Second, maternal preeclampsia was physician diagnosed and was extracted from the electronic medical records. Third, we had a large sample size with a median of 7 SBP observations per child, thus minimizing measurement error.^55^ Fourth, our sample predominantly comprised underrepresented (62% Black, 19% Hispanic) mothers and preterm birth children (19%); thus our findings are directly relevant to this important segment of the population.

This study has limitations. First, as an observational study, unmeasured confounding may exist. This is particularly true with respect to maternal nutritional factors (eg, protein and serum uric acid level) and certain lifestyle factors such as sunlight exposure which are difficult to measure and may change over the seasons of the year. However, in sensitivity analyses, we did not find that seasonality altered our associations. Low cord blood 25(OH)D levels may also reflect a poorer maternal health status in general which may predispose their children to higher BP. In this analysis, we did adjust for a comprehensive set of confounders including educational level as a measure of socioeconomic status, which has also been associated with diet quality and lifestyle.^56,57^ Second, we did not have data on the onset time of preeclampsia and were thus not able to distinguish the effects of early- vs late-onset of preeclampsia that may differ in pathogenesis and pathophysiology.^58,59^ Nevertheless, children born to women with early-onset of preeclampsia are more likely to have younger gestational age at birth^60^ and we did not find the associations examined in this analysis differ by preterm birth status. Third, we did not measure vitamin D concentration in maternal blood in our study. Fourth, a small proportion of children (n = 67 [8.9%]) only had 1 SBP measured during follow up.

## Conclusions

This study found maternal preeclampsia to be associated with higher child BP from early childhood to adolescence. Adequate cord blood 25(OH)D levels may modify this association. For mothers who experience preeclampsia during pregnancy, optimizing vitamin D levels may help prevent their children from developing high BP and future cardiovascular diseases. Future prospective birth cohorts and clinical trials are needed to confirm this benefit.

## Notes

Supplement. **eFigure 1.** Diagram of the Participant Selection Process for the Analysis (n=754) **eFigure 2.** Forest Plot of Change in Mean Systolic Blood Pressure Percentile per 5 ng/mL Increment in Cord Blood 25(OH)D Level Among Children Born to Mothers with Preeclampsia by Child Developmental Stage, Sex, Maternal Race/ethnicity, Preterm Birth, Low Birth Weight, and Maternal Pre-pregnancy Weight Status **eTable 1.** Characteristics of the Mother-Child Pairs in this Analysis by Maternal Preeclampsia Status (n=754) **eTable 2.** Characteristics of the Mother-Child Pairs in this Analysis by Quartiles of Cord Blood 25(OH)D Level (n=754) **eTable 3.** Associations of Maternal Preeclampsia and Child Systolic Blood Pressure Percentile, Overall and by Child Developmental Stage, Sex, Maternal Race/ethnicity, Preterm Birth, Low Birth Weight, and Maternal Pre-pregnancy Weight Status (n=754) **eTable 4.** Associations of Maternal Preeclampsia and Child Systolic Blood Pressure Percentile From 3 to 18 Years of Age by Cord Blood 25(OH)D Concentrations (n=754) **eTable 5.** Sensitivity Analyses Examining whether Child Postnatal 25(OH)D Confounded and/or Modified the Association of Maternal Preeclampsia and Child Systolic Blood Pressure (n=586) **eTable 6.** Characteristics of the Mother-Child Pairs Included in This Analysis (n=754) vs Excluded From This Analysis

## References

[1] Steegers EA, von Dadelszen P, Duvekot JJ, Pijnenborg R Pre-eclampsia. Lancet. 2010;376(9741):631-644. doi:10.1016/S0140-6736(10)60279-6 20598363

[2] Wallis AB, Saftlas AF, Hsia J, Atrash HK Secular trends in the rates of preeclampsia, eclampsia, and gestational hypertension, United States, 1987-2004. Am J Hypertens. 2008;21(5):521-526. doi:10.1038/ajh.2008.20 18437143

[3] Rosner B, Cook NR, Daniels S, Falkner B Childhood blood pressure trends and risk factors for high blood pressure: the NHANES experience 1988-2008. Hypertension. 2013;62(2):247-254. doi:10.1161/HYPERTENSIONAHA.111.00831 23856492 PMC3769135

[4] Chen X, Wang Y Tracking of blood pressure from childhood to adulthood: a systematic review and meta-regression analysis. Circulation. 2008;117(25):3171-3180. doi:10.1161/CIRCULATIONAHA.107.730366 18559702 PMC3568631

[5] Yang L, Magnussen CG, Yang L, Bovet P, Xi B Elevated Blood Pressure in Childhood or Adolescence and Cardiovascular Outcomes in Adulthood: A Systematic Review. Hypertension. 2020;75(4):948-955. doi:10.1161/HYPERTENSIONAHA.119.14168 32114851

[6] Davis EF, Lazdam M, Lewandowski AJ, Cardiovascular risk factors in children and young adults born to preeclamptic pregnancies: a systematic review. Pediatrics. 2012;129(6):e1552-e1561. doi:10.1542/peds.2011-3093 22614768

[7] Stark MJ, Clifton VL, Wright IMR Neonates born to mothers with preeclampsia exhibit sex-specific alterations in microvascular function. Pediatr Res. 2009;65(3):292-295. doi:10.1203/PDR.0b013e318193edf1 19391250

[8] Schalekamp-Timmermans S, Arends LR, Alsaker E, ; Global Pregnancy Collaboration Fetal sex-specific differences in gestational age at delivery in pre-eclampsia: a meta-analysis. Int J Epidemiol. 2017;46(2):632-642. 27605586 10.1093/ije/dyw178 PMC5837300

[9] Michos ED, Melamed ML Vitamin D and cardiovascular disease risk. Curr Opin Clin Nutr Metab Care. 2008;11(1):7-12. doi:10.1097/MCO.0b013e3282f2f4dd 18090651

[10] Fogacci S, Fogacci F, Banach M, ; Lipid and Blood Pressure Meta-analysis Collaboration (LBPMC) Group Vitamin D supplementation and incident preeclampsia: A systematic review and meta-analysis of randomized clinical trials. Clin Nutr. 2020;39(6):1742-1752. doi:10.1016/j.clnu.2019.08.015 31526611

[11] Wagner CL, Hollis BW The Implications of Vitamin D Status During Pregnancy on Mother and her Developing Child. Front Endocrinol (Lausanne). 2018;9:500-500. doi:10.3389/fendo.2018.00500 30233496 PMC6127214

[12] Wang G, Liu X, Bartell TR, Pearson C, Cheng TL, Wang X Vitamin D Trajectories From Birth to Early Childhood and Elevated Systolic Blood Pressure During Childhood and Adolescence. Hypertension. 2019;74(2):A11913120. doi:10.1161/HYPERTENSIONAHA.119.13120 31256718 PMC6938578

[13] Sauder KA, Stamatoiu AV, Leshchinskaya E, Ringham BM, Glueck DH, Dabelea D Cord Blood Vitamin D Levels and Early Childhood Blood Pressure: The Healthy Start Study. J Am Heart Assoc. 2019;8(9):e011485. doi:10.1161/JAHA.118.011485 31020895 PMC6512116

[14] Larsen SD, Dalgård C, Christensen ME, Blood pressure in 3-year-old girls associates inversely with umbilical cord serum 25-hydroxyvitamin D: an Odense Child Cohort study. Endocr Connect. 2018;7(12):1236-1244. doi:10.1530/EC-18-0308 30533001 PMC6240151

[15] Wang G, Divall S, Radovick S, Preterm birth and random plasma insulin levels at birth and in early childhood. JAMA. 2014;311(6):587-596. doi:10.1001/jama.2014.1 24519298 PMC4392841

[16] ACOG Committee on Obstetric Practice; American College of Obstetricians and Gynecologists ACOG practice bulletin. Diagnosis and management of preeclampsia and eclampsia. Number 33, January 2002. Int J Gynaecol Obstet. 2002;77(1):67-75. 12094777

[17] Zhang M, Mueller NT, Wang H, Hong X, Appel LJ, Wang X Maternal Exposure to Ambient Particulate Matter ≤2.5 μm During Pregnancy and the Risk for High Blood Pressure in Childhood. Hypertension. 2018;72(1):194-201. doi:10.1161/HYPERTENSIONAHA.117.10944 29760154 PMC6002908

[18] Flynn JT, Kaelber DC, Baker-Smith CM, ; SUBCOMMITTEE ON SCREENING AND MANAGEMENT OF HIGH BLOOD PRESSURE IN CHILDREN Clinical Practice Guideline for Screening and Management of High Blood Pressure in Children and Adolescents. Pediatrics. 2017;140(3):e20171904. doi:10.1542/peds.2017-1904 28827377

[19] Saenger AK, Laha TJ, Bremner DE, Sadrzadeh SM Quantification of serum 25-hydroxyvitamin D(2) and D(3) using HPLC-tandem mass spectrometry and examination of reference intervals for diagnosis of vitamin D deficiency. Am J Clin Pathol. 2006;125(6):914-920. doi:10.1309/J32UF7GTQPWN25AP 16690491

[20] Institute of Medicine Standing Committee on the Scientific Evaluation of Dietary Reference I. The National Academies Collection: Reports funded by National Institutes of Health. Dietary Reference Intakes for Calcium, Phosphorus, Magnesium, Vitamin D, and Fluoride. National Academies Press (US) National Academy of Sciences; 1997. 23115811

[21] Liu X, Arguelles L, Zhou Y, Longitudinal trajectory of vitamin D status from birth to early childhood in the development of food sensitization. Pediatr Res. 2013;74(3):321-326. doi:10.1038/pr.2013.110 23797532 PMC3773018

[22] Liu X, Wang G, Hong X, Gene-vitamin D interactions on food sensitization: a prospective birth cohort study. Allergy. 2011;66(11):1442-1448. doi:10.1111/j.1398-9995.2011.02681.x 21819409 PMC3189275

[23] Ross AC, Manson JE, Abrams SA, The 2011 report on dietary reference intakes for calcium and vitamin D from the Institute of Medicine: what clinicians need to know. J Clin Endocrinol Metab. 2011;96(1):53-58. doi:10.1210/jc.2010-2704 21118827 PMC3046611

[24] VanderWeele TJ, Shpitser I On the definition of a confounder. Ann Stat. 2013;41(1):196-220. doi:10.1214/12-AOS1058 25544784 PMC4276366

[25] Shoben AB, Kestenbaum B, Levin G, Seasonal variation in 25-hydroxyvitamin D concentrations in the cardiovascular health study. Am J Epidemiol. 2011;174(12):1363-1372. doi:10.1093/aje/kwr258 22112344 PMC3276302

[26] White IR, Royston P, Wood AM Multiple imputation using chained equations: Issues and guidance for practice. Stat Med. 2011;30(4):377-399. doi:10.1002/sim.4067 21225900

[27] Howe CJ, Cole SR, Lau B, Napravnik S, Eron JJ Jr Selection Bias Due to Loss to Follow Up in Cohort Studies. Epidemiology. 2016;27(1):91-97. doi:10.1097/EDE.0000000000000409 26484424 PMC5008911

[28] Langford HG, Watson RL Prepregnant blood pressure, hypertension during pregnancy, and later blood pressure of mothers and offspring. Hypertension. 1980;2(4 Pt 2):130-133. doi:10.1161/01.HYP.2.4.130 7399645

[29] Palti H, Rothschild E Blood pressure and growth at 6 years of age among offsprings of mothers with hypertension of pregnancy. Early Hum Dev. 1989;19(4):263-269. doi:10.1016/0378-3782(89)90061-3 2806155

[30] Seidman DS, Laor A, Gale R, Stevenson DK, Mashiach S, Danon YL Pre-eclampsia and offspring’s blood pressure, cognitive ability and physical development at 17-years-of-age. Br J Obstet Gynaecol. 1991;98(10):1009-1014. doi:10.1111/j.1471-0528.1991.tb15339.x 1751432

[31] Vatten LJ, Romundstad PR, Holmen TL, Hsieh CC, Trichopoulos D, Stuver SO Intrauterine exposure to preeclampsia and adolescent blood pressure, body size, and age at menarche in female offspring. Obstet Gynecol. 2003;101(3):529-533. 12636958 10.1016/s0029-7844(02)02718-7

[32] Tenhola S, Rahiala E, Martikainen A, Halonen P, Voutilainen R Blood pressure, serum lipids, fasting insulin, and adrenal hormones in 12-year-old children born with maternal preeclampsia. J Clin Endocrinol Metab. 2003;88(3):1217-1222. doi:10.1210/jc.2002-020903 12629109

[33] Tenhola S, Rahiala E, Halonen P, Vanninen E, Voutilainen R Maternal preeclampsia predicts elevated blood pressure in 12-year-old children: evaluation by ambulatory blood pressure monitoring. Pediatr Res. 2006;59(2):320-324. doi:10.1203/01.pdr.0000196734.54473.e3 16439600

[34] Geelhoed JJM, Fraser A, Tilling K, Preeclampsia and gestational hypertension are associated with childhood blood pressure independently of family adiposity measures: the Avon Longitudinal Study of Parents and Children. Circulation. 2010;122(12):1192-1199. doi:10.1161/CIRCULATIONAHA.110.936674 20823385 PMC5321267

[35] Lawlor DA, Macdonald-Wallis C, Fraser A, Cardiovascular biomarkers and vascular function during childhood in the offspring of mothers with hypertensive disorders of pregnancy: findings from the Avon Longitudinal Study of Parents and Children. Eur Heart J. 2012;33(3):335-345. doi:10.1093/eurheartj/ehr300 21862461 PMC3270043

[36] Fraser A, Nelson SM, Macdonald-Wallis C, Sattar N, Lawlor DA Hypertensive disorders of pregnancy and cardiometabolic health in adolescent offspring. Hypertension. 2013;62(3):614-620. doi:10.1161/HYPERTENSIONAHA.113.01513 23918754 PMC3819520

[37] Kvehaugen AS, Dechend R, Ramstad HB, Troisi R, Fugelseth D, Staff AC Endothelial function and circulating biomarkers are disturbed in women and children after preeclampsia. Hypertension. 2011;58(1):63-69. doi:10.1161/HYPERTENSIONAHA.111.172387 21606387

[38] Belfort MB, Gillman MW, McCormick MC Prenatal and perinatal predictors of blood pressure at school age in former preterm, low birth weight infants. J Perinatol. 2012;32(4):265-269. doi:10.1038/jp.2011.88 21738122 PMC4638382

[39] Miettola S, Hartikainen A-L, Vääräsmäki M, Offspring’s blood pressure and metabolic phenotype after exposure to gestational hypertension in utero. Eur J Epidemiol. 2013;28(1):87-98. doi:10.1007/s10654-013-9763-5 23354981

[40] Staley JR, Bradley J, Silverwood RJ, Associations of blood pressure in pregnancy with offspring blood pressure trajectories during childhood and adolescence: findings from a prospective study. J Am Heart Assoc. 2015;4(5):e001422. doi:10.1161/JAHA.114.001422 25994439 PMC4599398

[41] Øglaend B, Forman MR, Romundstad PR, Nilsen ST, Vatten LJ Blood pressure in early adolescence in the offspring of preeclamptic and normotensive pregnancies. J Hypertens. 2009;27(10):2051-2054. doi:10.1097/HJH.0b013e328330052a 19609220

[42] Ji H, Kim A, Ebinger JE, Sex Differences in Blood Pressure Trajectories Over the Life Course. JAMA Cardiol. 2020;5(3):19-26. doi:10.1001/jamacardio.2019.5306 31940010 PMC6990675

[43] Xia S, Du X, Guo L, Sex Differences in Primary and Secondary Prevention of Cardiovascular Disease in China. Circulation. 2020;141(7):530-539. doi:10.1161/CIRCULATIONAHA.119.043731 32065775

[44] Alexander BT, Dasinger JH, Intapad S Fetal programming and cardiovascular pathology. Compr Physiol. 2015;5(2):997-1025. doi:10.1002/cphy.c140036 25880521 PMC4772789

[45] Davis EF, Newton L, Lewandowski AJ, Pre-eclampsia and offspring cardiovascular health: mechanistic insights from experimental studies. Clin Sci (Lond). 2012;123(2):53-72. doi:10.1042/CS20110627 22455350 PMC3315178

[46] Jayet P-Y, Rimoldi SF, Stuber T, Pulmonary and systemic vascular dysfunction in young offspring of mothers with preeclampsia. Circulation. 2010;122(5):488-494. doi:10.1161/CIRCULATIONAHA.110.941203 20644018

[47] Gray KJ, Kovacheva VP, Mirzakhani H, Gene-Centric Analysis of Preeclampsia Identifies Maternal Association at *PLEKHG1.* Hypertension. 2018;72(2):408-416. doi:10.1161/HYPERTENSIONAHA.117.10688 29967039 PMC6043396

[48] Williams PJ, Broughton Pipkin F The genetics of pre-eclampsia and other hypertensive disorders of pregnancy. Best Pract Res Clin Obstet Gynaecol. 2011;25(4):405-417. doi:10.1016/j.bpobgyn.2011.02.007 21429808 PMC3145161

[49] Norman PE, Powell JT Vitamin D and cardiovascular disease. Circ Res. 2014;114(2):379-393. doi:10.1161/CIRCRESAHA.113.301241 24436433

[50] Al Mheid I, Quyyumi AA Vitamin D and Cardiovascular Disease: Controversy Unresolved. J Am Coll Cardiol. 2017;70(1):89-100. doi:10.1016/j.jacc.2017.05.031 28662812

[51] O’Connell TD, Berry JE, Jarvis AK, Somerman MJ, Simpson RU 1,25-Dihydroxyvitamin D3 regulation of cardiac myocyte proliferation and hypertrophy. Am J Physiol. 1997;272(4 Pt 2):H1751-H1758. doi:10.1152/ajpheart.1997.272.4.H1751 9139959

[52] Li YC, Kong J, Wei M, Chen ZF, Liu SQ, Cao LP 1,25-Dihydroxyvitamin D(3) is a negative endocrine regulator of the renin-angiotensin system. J Clin Invest. 2002;110(2):229-238. doi:10.1172/JCI0215219 12122115 PMC151055

[53] Murthi P, Yong HE, Ngyuen TP, Role of the Placental Vitamin D Receptor in Modulating Feto-Placental Growth in Fetal Growth Restriction and Preeclampsia-Affected Pregnancies. Front Physiol. 2016;7:43. doi:10.3389/fphys.2016.00043 26924988 PMC4757640

[54] Bodnar LM, Catov JM, Simhan HN, Holick MF, Powers RW, Roberts JM Maternal vitamin D deficiency increases the risk of preeclampsia. J Clin Endocrinol Metab. 2007;92(9):3517-3522. doi:10.1210/jc.2007-0718 17535985 PMC4288954

[55] Hutcheon JA, Chiolero A, Hanley JA Random measurement error and regression dilution bias. BMJ. 2010;340:c2289. doi:10.1136/bmj.c2289 20573762

[56] Darmon N, Drewnowski A Does social class predict diet quality? Am J Clin Nutr. 2008;87(5):1107-1117. doi:10.1093/ajcn/87.5.1107 18469226

[57] Wang J, Geng L Effects of Socioeconomic Status on Physical and Psychological Health: Lifestyle as a Mediator. Int J Environ Res Public Health. 2019;16(2):281. doi:10.3390/ijerph16020281 30669511 PMC6352250

[58] Raymond D, Peterson E A critical review of early-onset and late-onset preeclampsia. Obstet Gynecol Surv. 2011;66(8):497-506. doi:10.1097/OGX.0b013e3182331028 22018452

[59] Liu T, Zhang M, Guallar E, Trace Minerals, Heavy Metals, and Preeclampsia: Findings from the Boston Birth Cohort. J Am Heart Assoc. 2019;8(16):e012436. doi:10.1161/JAHA.119.012436 31426704 PMC6759885

[60] Odegård RA, Vatten LJ, Nilsen ST, Salvesen KA, Austgulen R Preeclampsia and fetal growth. Obstet Gynecol. 2000;96(6):950-955. 11084184

