# Supplementary material for: Associations of Cord Blood Vitamin D and Preeclampsia With Offspring Blood Pressure in Childhood and Adolescence
Source: JAMA Netw Open. 2020 Oct 5;3(10):e2019046. doi: 10.1001/jamanetworkopen.2020.19046 (PMC7536588; doi:10.1001/jamanetworkopen.2020.19046)
Supplement: Supplement. — eFigure 1. Diagram of the Participant Selection Process for the Analysis (n=754) eFigure 2. Forest Plot of Change in Mean Systolic Blood Pressure Percentile per 5 ng/mL Increment in Cord Blood 25(OH)D Level Among Children Born to Mothers with Preeclampsia by Child Developmental Stage, Sex, Maternal Race/ethnicity, Preterm Birth, Low Birth Weight, and Maternal Pre-pregnancy Weight Status eTable 1. Characteristics of the Mother-Child Pairs in this Analysis by Maternal Preeclampsia Status (n=754) eTable 2. Characteristics of the Mother-Child Pairs in this Analysis by Quartiles of Cord Blood 25(OH)D Level (n=754) eTable 3. Associations of Maternal Preeclampsia and Child Systolic Blood Pressure Percentile, Overall and by Child Developmental Stage, Sex, Maternal Race/ethnicity, Preterm Birth, Low Birth Weight, and Maternal Pre-pregnancy Weight Status (n=754) eTable 4. Associations of Maternal Preeclampsia and Child Systolic Blood Pressure Percentile From 3 to 18 Years of Age by Cord Blood 25(OH)D Concentrations (n=754) eTable 5. Sensitivity Analyses Examining whether Child Postnatal 25(OH)D Confounded and/or Modified the Association of Maternal Preeclampsia and Child Systolic Blood Pressure (n=586) eTable 6. Characteristics of the Mother-Child Pairs Included in This Analysis (n=754) vs Excluded From This Analysis [file jamanetwopen-e2019046-s001.pdf]

Supplemental Online Content

Zhang M, Michos ED, Wang G, Wang X, Mueller NT. Associations of cord blood vitamin D and preeclampsia with offspring blood pressure in childhood and adolescence. *JAMA Netw Open*. 2020;3(10):e2019046. doi:10.1001/jamanetworkopen.2020.19046

eFigure 1. Diagram of the Participant Selection Process for the Analysis (n=754)
eFigure 2. Forest Plot of Change in Mean Systolic Blood Pressure Percentile per 5 ng/mL Increment in Cord Blood 25(OH)D Level Among Children Born to Preeclamptic Mothers by Child Developmental Stage, Sex, Race/ethnicity, Preterm Birth, Low Birthweight, and Maternal Pre-pregnancy Weight Status
eTable 1. Characteristics of the Mother-Child Pairs in this Analysis by Maternal Preeclampsia Status (n=754)
eTable 2. Characteristics of the Mother-Child Pairs in this Analysis by Quartiles of Cord Blood 25(OH)D Level (n=754)
eTable 3. Associations of Maternal Preeclampsia and Child Systolic Blood Pressure Percentile, Overall and by Child Developmental Stage, Sex, Race/ethnicity, Preterm Birth, Low Birthweight, and Maternal Pre-pregnancy Weight Status (n=754)
eTable 4. Associations of Maternal Preeclampsia and Child Systolic Blood Pressure Percentile From 3 to 18 Years of Age by Cord Blood 25(OH)D Concentrations (n=754)
eTable 5. Sensitivity Analyses Examining if Child Postnatal 25(OH)D Confounded and/or Modified the Association of Maternal Preeclampsia and Child Blood Pressure (n=586)
eTable 6. Characteristics of the Mother-Child Pairs Included in This Analysis (n=754) vs Excluded From This Analysis

This supplemental material has been provided by the authors to give readers additional information about their work.

© 2020 Zhang M et al. *JAMA Network Open*.

eFigure 1. Diagram of the participant selection process for the analysis (*n*=754)

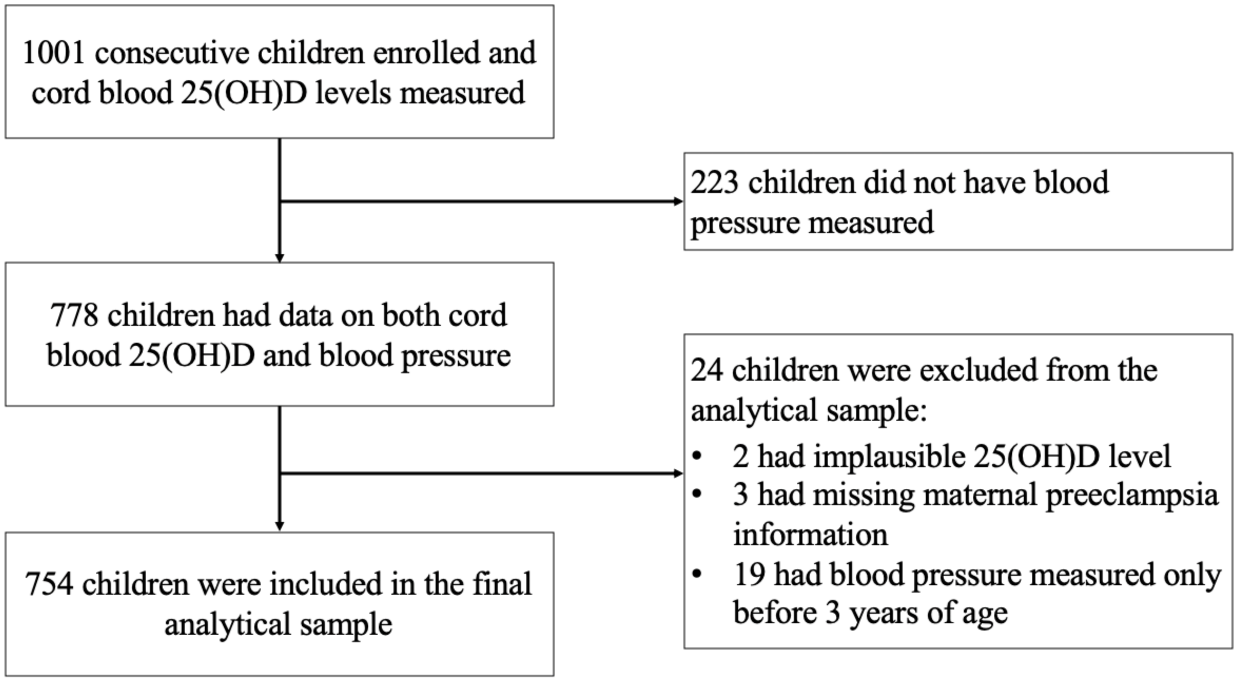

© 2020 Zhang M et al. *JAMA Network Open*.

eFigure 2. **Forest plot of change in mean systolic blood pressure percentile per 5 ng/mL increment in cord blood 25(OH)D level among children born to preeclamptic mothers by child developmental stage, sex, race/ethnicity, preterm birth, low birthweight, and maternal pre-pregnancy weight status.** Models adjusted for maternal age at delivery, race/ethnicity (if not stratified by race/ethnicity), educational achievement, smoking status during pregnancy, and maternal pre-pregnancy BMI (if not stratified by maternal BMI). BP indicates blood pressure; BMI, body mass index; CI, confidence interval.

Groups	No. of children	Change in mean systolic blood pressure percentile per 5 ng/mL increment in cord blood 25(OH)D among children born to preeclamptic mothers: Adjusted β* (95% CI)	Forest plot	*P* for interaction
Overall	754	-3.47 (-6.18, -0.77)		N/A
Early childhood (3 to 5 years)	672	-3.90 (-7.21, -0.60)		0.01
Middle childhood (6 to 12 years)	650	-2.84 (-5.77, 0.10)		
Adolescence (13 to 18 years)	143	-7.80 (-12.30, -3.29)		
Female	377	-5.81 (-9.13, -2.49)		0.05
Male	377	1.00 (-3.53, 5.54)		
Black	469	-3.82 (-6.84, -0.80)		0.67
Hispanic	144	-2.16 (-13.47, 9.14)		
Term birth	614	-4.71 (-8.34, -1.07)		0.72
Preterm birth	140	-1.95 (-5.92, 2.02)		
Normal birth weight	634	-2.98 (-6.12, 0.17)		0.81
Low birth weight	120	-4.89 (-10.02, 0.25)		
Maternal BMI 18.5-24.9 kg/m^2	298	-4.21 (-9.73, 1.31)		0.55
Maternal BMI ≥ 25 kg/m^2	396	-2.23 (-5.62, 1.15)		

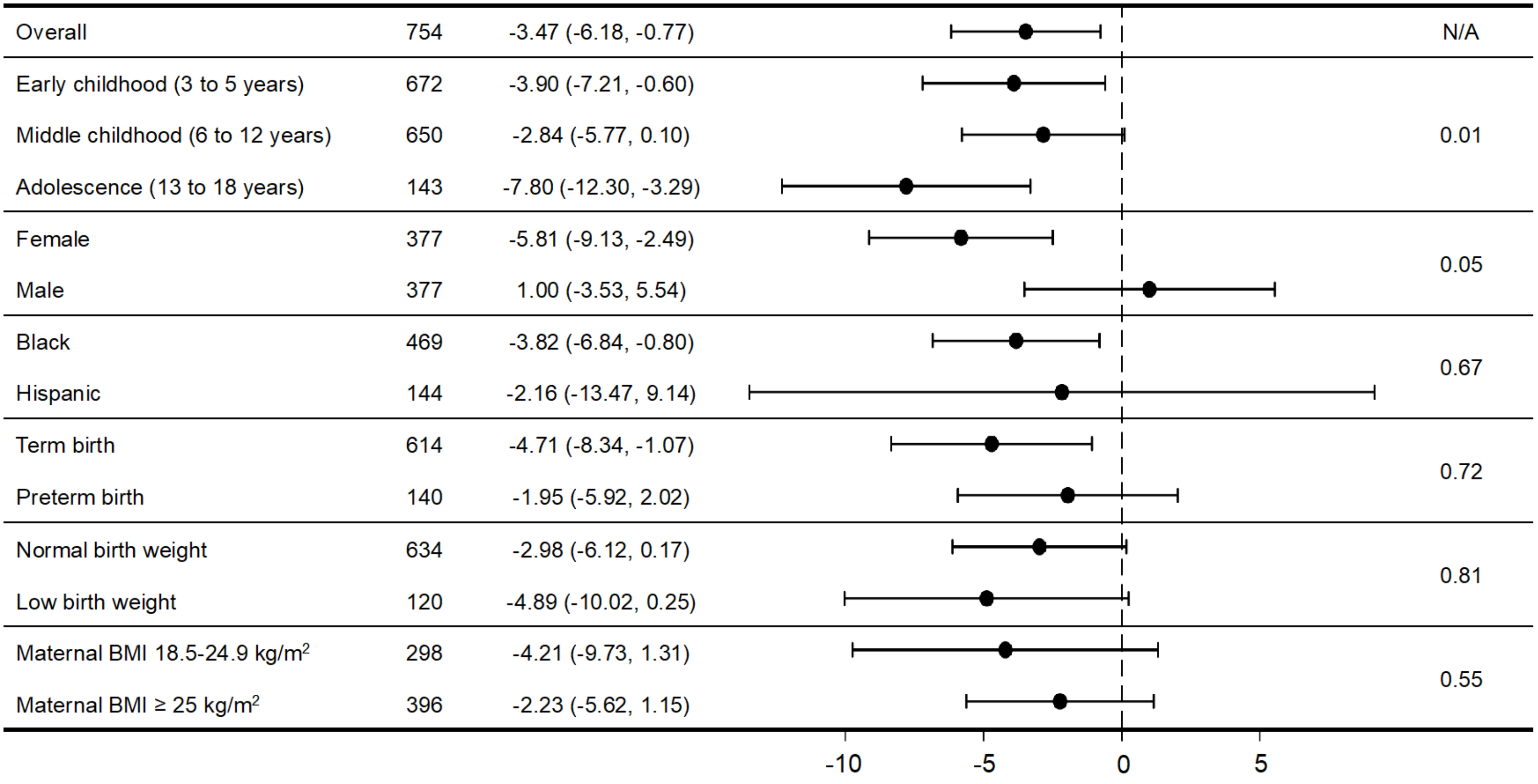

© 2020 Zhang M et al. *JAMA Network Open*.

eTable 1. **Characteristics of the mother-child pairs in this analysis by maternal preeclampsia status (*n*=754)**

Characteristic, n (%)*	No Preeclampsia	Preeclampsia
N	675	79
**Maternal characteristics**		
Age at delivery, years, mean (SD)	28.5 (6.7)	29.9 (6.6)
Black	415 (61.5%)	54 (68.4%)
Married	224 (33.2%)	27 (34.2%)
Did not finish high school	198 (29.3%)	21 (26.6%)
Smoked during pregnancy	64 (9.5%)	12 (15.2%)
Pre-pregnancy body mass index, kg/m^2, mean (SD)	26.6 (6.3)	29.7 (6.3)
Overweight or obese	339 (50.2%)	57 (72.2%)
**Child characteristics**		
Number of blood pressure measurements, median (IQR)	7.0 (4.0-11.0)	8.0 (3.0-11.0)
Cord blood 25(OH)D concentration, ng/mL, median (IQR)	12.2 (7.9-17.2)	12.1 (8.1-17.2)
Female	334 (49.5%)	43 (54.4%)
Gestational age, weeks, mean (SD)	38.7 (2.3)	36.8 (2.8)
Preterm birth	102 (15.1%)	38 (48.1%)
Birthweight, grams, mean (SD)	3151.9 (610.1)	2692.8 (825.4)
Low birth weight	86 (12.7%)	34 (43.0%)

* Unless otherwise indicated

SD indicates standard deviation; IQR, interquartile range.

© 2020 Zhang M et al. *JAMA Network Open*.

eTable 2. **Characteristics of the mother-child pairs in this analysis by quartiles of cord blood 25(OH)D level (*n*=754)**

Variable, n (%)*	Quartile 1 (1.4-7.9 ng/mL)	Quartile 2 (8.0-12.2 ng/mL)	Quartile 3 (12.3-17.1 ng/mL)	Quartile 4 (17.2-73.5 ng/mL)
N	190	187	188	189
**Maternal characteristics**				
Age at delivery, years, mean (SD)	27.2 (6.5)	28.9 (6.5)	29.0 (6.7)	29.5 (7.0)
Black	146 (76.8%)	119 (63.6%)	115 (61.2%)	89 (47.1%)
Married	44 (23.2%)	74 (39.6%)	65 (34.6%)	68 (36.0%)
Did not finish high school	54 (28.4%)	50 (26.7%)	55 (29.3%)	60 (31.7%)
Smoked during pregnancy	25 (13.2%)	12 (6.4%)	18 (9.6%)	21 (11.1%)
Pre-pregnancy body mass index, kg/m^2, mean (SD)	27.5 (6.6)	27.4 (7.2)	26.6 (5.9)	26.1 (5.4)
Overweight or obese	104 (54.7%)	106 (56.7%)	97 (51.6%)	89 (47.1%)
**Child characteristics**				
Number of blood pressure measurements, median (IQR)	8.0 (4.0-11.0)	7.0 (4.0-12.0)	6.0 (3.0-11.0)	7.0 (4.0-11.0)
Cord blood 25(OH)D concentration, ng/mL, median (IQR)	5.8 (4.7-6.8)	9.9 (8.9-11.2)	14.5 (13.4-15.9)	22.3 (19.2-26.4)
Female	95 (50.0%)	87 (46.5%)	93 (49.5%)	102 (54.0%)
Gestational age, weeks, mean (SD)	38.6 (2.1)	38.6 (2.4)	38.2 (2.7)	38.6 (2.7)
Preterm birth	29 (15.3%)	34 (18.2%)	38 (20.2%)	39 (20.6%)
Birthweight, grams, mean (SD)	3116.2 (595.9)	3100.0 (613.4)	3038.1 (702.4)	3160.6 (684.7)
Low birth weight	24 (12.6%)	25 (13.4%)	38 (20.2%)	33 (17.5%)

* Unless otherwise indicated

SD indicates standard deviation; IQR, interquartile range.

© 2020 Zhang M et al. *JAMA Network Open*.

eTable 3. **Associations of maternal preeclampsia and child systolic blood pressure percentile, overall and by child developmental stage, sex, race/ethnicity, preterm birth, low birthweight, and maternal pre-pregnancy weight status (*n*=754)**

Groups	No. of children	No. of blood pressure observations	Average child systolic blood pressure percentile comparing those born to preeclamptic mothers vs. non-preeclamptic mothers				
			Crude β (95% CI)	Adjusted β (95% confidence interval)			
				Adjusted for confounders[1]	Additionally adjusted for season of delivery[2]	Applied SIPW weight[1]	Applied MICE method[1]
**Overall**	754	6,669	5.75 (1.78, 9.71)	5.34 (1.37, 9.30)	5.32 (1.35, 9.28)	5.78 (1.99, 9.58)	5.41 (1.43, 9.39)
**Developmental stage**							
Early childhood (3 to 5 years)	672	1,753	3.60 (-1.26, 8.47)	3.25 (-1.62, 8.12)	3.20 (-1.66, 8.07)	3.79 (-0.91, 8.48)	3.31 (-1.57, 8.19)
Middle childhood (6 to 12 years)	650	4,265	6.52 (2.19, 10.85)	6.15 (1.82, 10.47)	6.14 (1.81, 10.46)	6.70 (1.87, 11.53)	6.19 (1.85, 10.53)
Adolescent (13 to 18 years)	143	651	11.11 (2.84, 19.37)	10.81 (2.55, 19.08)	10.84 (2.57, 19.10)	10.87 (-3.14, 24.87)	10.95 (2.68, 19.22)
**Child sex**							
Female	377	3,023	6.68 (1.22, 12.15)	6.64 (1.21, 12.08)	6.61 (1.17, 12.05)	6.79 (1.10, 12.49)	6.65 (1.20, 12.10)
Male	377	3,646	4.56 (-1.19, 10.30)	3.77 (-1.96, 9.50)	3.77 (-1.96, 9.50)	4.55 (-0.20, 9.30)	3.90 (-1.85, 9.64)
**Race/Ethnicity**							
Black	469	4,057	4.23 (-0.56, 9.01)	3.70 (-1.07, 8.47)	3.77 (-1.00, 8.54)	4.11 (-0.38, 8.61)	3.76 (-1.02, 8.55)
Hispanic	144	1,342	17.42 (6.08, 28.77)	16.46 (5.11, 27.81)	15.89 (4.51, 27.26)	16.59 (8.91, 24.27)	16.87 (5.53, 28.22)
**Preterm birth**							
Term birth	614	5,169	5.37 (0.02, 10.71)	4.89 (-0.41, 10.19)	4.70 (-0.61, 10.01)	5.11 (-0.05, 10.27)	4.74 (-0.58, 10.06)
Preterm birth	140	1,500	3.43 (-2.90, 9.77)	3.07 (-3.27, 9.40)	3.28 (-3.06, 9.62)	4.12 (-1.87, 10.11)	3.40 (-2.93, 9.73)

© 2020 Zhang M et al. *JAMA Network Open*.

**Low birth weight**							
Normal birthweight	634	5,381	4.95 (-0.18, 10.08)	4.77 (-0.34, 9.87)	4.74 (-0.36, 9.84)	5.47 (0.56, 10.38)	4.74 (-0.39, 9.87)
Low birthweight	120	1,288	4.16 (-2.58, 10.91)	3.41 (-3.28, 10.09)	3.50 (-3.19, 10.18)	3.74 (-2.49, 9.97)	3.66 (-3.02, 10.34)
**Maternal overweight or obese**							
Maternal BMI 18.5-24.9 kg/m^2	298	2,558	8.35 (0.39, 16.32)	8.30 (0.41, 16.20)	8.35 (0.46, 16.24)	8.76 (0.88, 16.63)	8.16 (0.26, 16.06)
Maternal BMI ≥ 25 kg/m^2	396	3,509	4.93 (0.16, 9.71)	4.91 (0.18, 9.64)	4.86 (0.14, 9.58)	5.35 (0.96, 9.74)	4.89 (0.16, 9.63)

[1] Models adjusted for maternal age at delivery, race/ethnicity, educational achievement, smoking status during pregnancy, and maternal pre-pregnancy body mass index.

[2] Models adjusted for maternal age at delivery, race/ethnicity, educational achievement, smoking status during pregnancy, maternal pre-pregnancy body mass index, and season of delivery.

BMI indicates body mass index; SIPW, stabilized inverse probability weighting; MICE, multiple imputation by chained equation.

© 2020 Zhang M et al. *JAMA Network Open*.

eTable 4. **Associations of maternal preeclampsia and child systolic blood pressure percentile from 3 to 18 years of age by cord blood 25(OH)D concentrations (*n*=754)**

Groups	No. of children	No. of blood pressure observations	Average child systolic blood pressure percentile comparing those born to preeclamptic mothers vs. non-preeclamptic mothers				
			Crude β (95% CI)	Adjusted β (95% confidence interval)			
				Adjusted for confounders[1]	Additionally adjusted for season of delivery[2]	Applied SIPW weight[1]	Applied MICE method[1]
Overall	754	6,669	5.75 (1.78, 9.71)	5.34 (1.37, 9.30)	5.32 (1.35, 9.28)	5.78 (1.99, 9.58)	5.41 (1.43, 9.39)
By Vitamin D deficiency (cord blood 25(OH)D < 11 ng/mL)							
No	324	3,618	4.32 (-0.84, 9.48)	3.71 (-1.46, 8.87)	3.70 (-1.46, 8.87)	4.17 (-1.04, 9.37)	3.69 (-1.49, 8.87)
Yes	430	3,051	7.80 (1.61, 14.00)	7.73 (1.60, 13.86)	7.70 (1.57, 13.83)	8.18 (2.93, 13.43)	7.94 (1.80, 14.09)
By Vitamin D deficiency (sensitivity analysis: cord blood 25(OH)D < 20 ng/mL)							
No	128	1,066	-3.98 (-13.07, 5.12)	-4.19 (-13.26, 4.88)	-4.18 (-13.24, 4.88)	-3.76 (-13.81, 6.28)	-4.38 (-13.47, 4.71)
Yes	626	5,603	7.80 (3.61, 12.38)	7.49 (3.13, 11.85)	7.47 (3.12, 11.82)	7.92 (4.11, 11.73)	7.63 (3.26, 12.00)
By quartiles of cord blood 25(OH)D concentration							
Quartile 1 (1.4 to 7.9 ng/mL)	190	1,819	10.67 (2.56, 18.78)	10.56 (2.55, 18.56)	10.53 (2.53, 18.53)	11.01 (4.57, 17.46)	10.68 (2.65, 18.71)
Quartile 2 (8.0 to 12.2 ng/mL)	187	1,747	7.36 (-0.20, 14.92)	7.36 (-0.17, 14.88)	7.21 (-0.32, 14.74)	7.66 (0.92, 14.39)	7.55 (0.03, 15.08)
Quartile 3 (12.3 to 17.1 ng/mL)	188	1,546	6.04 (-2.03, 14.11)	4.94 (-3.07, 12.96)	4.98 (-3.05, 13.01)	5.40 (-1.69, 12.49)	5.00 (-3.03, 13.04)
Quartile 4 (17.2 to 73.5 ng/mL)	189	1,557	-1.22 (-9.04, 6.59)	-1.87 (-9.71, 5.96)	-1.80 (-9.63, 6.03)	-1.34 (-9.83, 7.15)	-2.00 (-9.85, 5.85)

[1] Models adjusted for maternal age at delivery, race/ethnicity, educational achievement, smoking status during pregnancy, and maternal pre-pregnancy body mass index.
[2] Models adjusted for maternal age at delivery, race/ethnicity, educational achievement, smoking status during pregnancy, maternal pre-pregnancy body mass index, and season of delivery.
SIPW indicates stabilized inverse probability weighting; MICE, multiple imputation by chained equation.

© 2020 Zhang M et al. *JAMA Network Open*.

eTable 5. **Sensitivity analyses examining if child postnatal 25(OH)D confounded and/or modified the association of maternal preeclampsia and child blood pressure (*n*=586)** Estimates show the average child systolic blood pressure percentile comparing those born to preeclamptic mothers vs. non-preeclamptic mothers (reference).

**Does postnatal 25(OH)D confound the association of maternal preeclampsia and child blood pressure?**		
Overall sample (*n*=754)	Subsample with postnatal 25(OH)D measurement (*n*=586)	
Adjusted for confounders[1]	Adjusted for confounders[1]	Additionally adjusted for postnatal 25(OH)D[1]
5.34 (1.37, 9.30)	6.69 (2.22, 11.16)	6.73 (2.26, 11.19)

**Does postnatal 25(OH)D modify the association of maternal preeclampsia and child blood pressure?**		
Quartiles of postnatal 25(OH)D	Adjusted for confounders[1]	*P* for interaction
Overall Sample (*n*=754)	5.34 (1.37, 9.30)	N/A
Quartile 1: 3.5 to 25.4 ng/mL (*n*=145)	12.76 (3.85, 21.69)	0.136
Quartile 2: 25.6 to 32.6 ng/mL (*n*=148)	-0.05 (-8.44, 8.34)	
Quartile 3: 32.7 to 39.0 ng/mL (*n*=147)	12.02 (3.24, 20.80)	
Quartile 4: 39.1 to 71.7 ng/mL (*n*=146)	4.52 (-4.64, 13.68)	

[1] Models adjusted for maternal age at delivery, race/ethnicity, educational achievement, smoking status during pregnancy, and maternal pre-pregnancy body mass index.
[2] Models adjusted for maternal age at delivery, race/ethnicity, educational achievement, smoking status during pregnancy, maternal pre-pregnancy body mass index, and child postnatal 25(OH)D

© 2020 Zhang M et al. *JAMA Network Open*.

eTable 6. **Characteristics of the mother-child pairs included this analysis (*n*=754) vs. excluded from this analysis (*n*=223)**

Variable, n (%)[*]	Included	Excluded
N	754	223
**Maternal characteristics**		
Age at delivery, years, mean (SD)	28.7 (6.7)	28.0 (5.8)
Black	469 (62.2%)	86 (38.6%)
Married	251 (33.3%)	77 (34.5%)
Did not finish high school	219 (29.0%)	63 (28.3%)
Smoked during pregnancy	76 (10.1%)	29 (13.0%)
Pre-pregnancy body mass index, kg/m^2, mean (SD)	26.9 (6.3)	24.9 (5.3)
Overweight or obese	396 (52.5%)	85 (38.1%)
**Child characteristics**		
Cord blood 25(OH)D concentration, ng/mL, median (IQR)	12.2 (7.9-17.2)	14.0 (9.3-19.1)
Female	377 (50.0%)	106 (47.5%)
Gestational age, weeks, mean (SD)	38.5 (2.5)	38.2 (3.1)
Preterm birth	140 (18.6%)	58 (26.0%)
Birthweight, grams, mean (SD)	3103.8 (650.8)	3030.0 (715.1)
Low birth weight	120 (15.9%)	44 (19.7%)

* Unless otherwise indicated

SD indicates standard deviation; IQR, interquartile range.

© 2020 Zhang M et al. *JAMA Network Open*.
